# Synthesis Based on a Preceramic Polymer and Alumina Nanoparticles via UV Lithography for High Temperature Applications

**DOI:** 10.3390/ma13051140

**Published:** 2020-03-04

**Authors:** Mohammed S. Almeataq, Eid M. Alosime

**Affiliations:** King Abdulaziz City for Science and Technology, P.O. Box 6086, Riyadh 11442, Saudi Arabia; mmeataq@kacst.edu.sa

**Keywords:** polymer derived ceramic, alumina, UV lithography, pyrolysis

## Abstract

Because of the increased demand for preceramic polymers in high-tech applications, there has been growing interest in the synthesis of preceramic polymers, including polysiloxanes and alumina. These polymers are preferred because of their low thermal expansion, conformability to surfaces over large areas, and flexibility. The primary objective was to evaluate the aspects of polymer-derived ceramic routs, focusing on the UV lithography process of preceramic polymers and the pyrolyzing properties of the final ceramics. We found that the p(DMS-co-AMS) copolymer was effective in scattering the hydrophilic Al_2_O_3_ nanoparticles into the exceedingly hydrophobic solvent. The physico-chemical behavior of characterized copolymers was explored during their pyrolytic transformation into amorphous silicon-based ceramics. The results indicate that an increase of the pyrolysis temperature degraded the Si–O network through the carbothermic reaction of silicon. We also found a rapid elimination of copolymer pores and densification when the temperature increased (1100 to 1200 °C). At different but specific temperature ranges, there are different distinct rearrangement reactions in the conversion of polymer to ceramic; reductions of the melting point (T_m_) of the total heat of melting (ΔH_m_) of the pyrolysis process resulted in the crystallization of ceramic materials; hence, lithography based on pyrolysis properties of preceramic polymers is a critical method in the conversation of polymers.

## 1. Introduction

The synthesis of preceramic polymers, such as polysiloxanes, has received much attention over the years because of polymers’ low thermal expansion, conformability to surfaces over large areas, and ability to obtain new binary ceramics, such as silicon carbide (SiC), and even more complex compositions in silicon oxycarbide (Si–OC) systems [1,2]. In addition, amending the starting polymer chemicals with metallic precursors, such as Al, Zr, and Ti, could provide a further increase in the complexity of the system, helping to realize ceramic components with a greater variety of compositions and outstanding thermal properties.

There are many reasons why the methodology is a potentially promising direction towards understanding ceramic components. These include the ability to achieve a wider variety of compositions, the unique microstructure characteristic which is often not associated with other conventional methods, the low cost of the precursors, and the fact that the final ceramics possess distinctive thermo-mechanical and chemical properties. Additionally, the inherent possibility of transforming the precursors using the polymer forming technologies (which may include injection molding, resin transfer molding, coating, polymer infiltration pyrolysis, fiber drawing, and injection molding). In the current work, the most crucial aspects of polymer derived ceramics (PDCs) will be discussed with a bias towards the UV lithography process behind the preceramic polymers and the characteristics of the final ceramics.

Polysiloxanes have very many applications, especially in high-tech fields. These materials are exceptionally useful in the aerospace industry in which semiconductor materials are utilized for making products such as silicon wafers. This class of polymers exhibits extremely interesting properties, and through continuing development, the polysiloxane market is expected to grow further, enabling additional novel applications, such as lithographic applications [3]. The synthesis, characterization, and application of ceramic materials synthesized through the controlled pyrolysis of preceramic polymers mixed with the fillers of a different nature have been identified as important for crystallization behavior [4]. One such approach for synthesizing is based on a technique called pyrolysis. In pyrolysis, nano-alumina particles combined with polysiloxane samples are warm-pressed (1500 °C) under an inert argon environment. As a result, the crystallization of mullite is initiated, which may also occur at 1300 °C. The nano-alumina particles provide magnified homogeneity in alumina nano-particle’s distribution within the polysiloxane. As such, the final product possesses a nanostructure composed of mullite crystals whose dimensions fall within 60–160 nm while those of SiC fall within the 1–8 nm range [4].

Of greater potential importance is the fact that mullite has been used as a resistant protective thermal barrier in very demanding applications, such as the heat shields for spacecraft. It was found that the ceramics obtained could be classified as bulk nanostructured ceramics and nano-composites; this has attracted remarkable attention in light of their interesting structural characteristics. Therefore, this presents a new approach for fabricating a preceramic polymer [5,6]. The prospective application of polysiloxane-derived Si–OC ceramics in the design of micro-electro-mechanical systems (MEMS) is also used.

Preceramic polymers are a type of polymer that can be converted into ceramics by a heat treatment typically above 800 °C [7]. Various methods can be used to process preceramic polymers, one of which is microcomponent processing using UV/electron beam lithography [8], a form of lithography using UV light or an electron beam as an illumination source to transfer a pattern into a substrate in the etching process. However, there is a shortage of comprehensive research on photolithography use in the processing of preceramic polymers, with most research instead focusing on microfluidic processing or soft lithography and PDCs [9,10].

According to delCampo and Arzt [11], research has mostly focused on trying to shrink the lateral dimensions of the imaged features. The authors suggested that the goal should be to improve shrinking to at least 45 nm by the year 2010, whereas research done on projection optical lithography by Rothschild [12] projected 32 nm nodes by 2013. Martínez-Crespiera et al. [13] tested the fabrication of Si–OC ceramic microcomponents from a preceramic polymer using photolithography and soft lithography, finding that the former had the advantage of allowing the production of microstructures on substrates; they also experimented with the use of photo-crosslinking as a procedure to obtain rapid and crack-free infusible polymers. This was highly successful because they achieved ratios as high as 3:1 and micro-sizes in the order of 20 µm.

Conventional photolithography, together with surface machining, is the most frequently used method to obtain the micrometer-sized surface features needed for sensors and actuators [14]. However, to remain competitive with traditional ceramics, preceramic polymers need to be inexpensive, and thus, the fabrication process needs to be held to a low cost [1,2]. Therefore, further research on the use of photolithography is required to make the process more economical. Overall, it is expected that the use of photolithography will continue, despite the numerous advancements in other preceramic polymer–processing techniques [11].

## 2. Experimental Section

### 2.1. Materials

Si wafers were purchased from Sigma-Aldrich (Saint Louis, MI, USA), polished to the (100) face, having a native oxide layer of approximately 15 Å thickness. Poly(dimethylsiloxane-co alkylmethylsiloxane) p(DMS-co-AMS) (Aldrich, Saint Louis, MI, USA); triphenylsulfonium triflate (TPST) (Aldrich, Saint Louis, MI, USA); aluminum oxide nanopowder (Aldrich, 99.8%, Saint Louis, MI, USA); toluene (Aldrich, 98%, Saint Louis, MI, USA); sulfuric acid (Certified ACS Plus); Fisher chemical (Suwanee, GA, USA); and hydrogen peroxide (Scharlau, 30%, Barcelona, Spain) were used. All other reagents were used as received from commercial sources.

### 2.2. Si Wafer Treatment

Here, 1 cm^2^ Si wafers were cleaned by immersing them in a so-called piranha solution, which is a solution of 30% H2O2:H2SO4 (v/v: 3/7), for 30 min. The wafers were then rinsed with copious amounts of deionized water several times and dried.

### 2.3. Experimental Procedure

**Figure 1:**
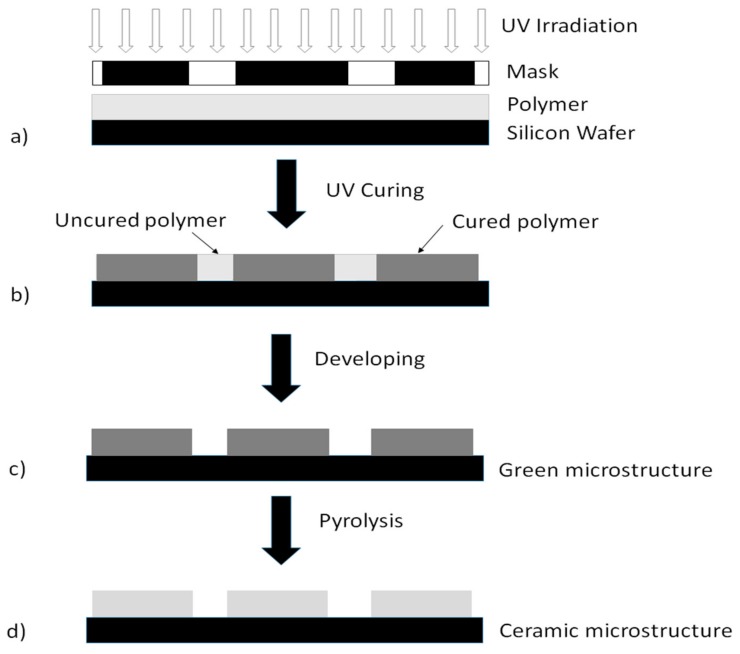
UV lithographic processing of p(PDMS-co-AMS) copolymers to produce SiOC-based microstructures on a silicon wafer. The polymer acts as a negative photoresist (black partitions of the mask manifest the holes). (**a** → **b**): UV curing of the polymer using a foil mask; (**b** → **c**): developing of the uncured polymer with solvents to obtain green microstructures on Si wafers; (**c** → **d**): pyrolysis step to produce the ceramic microstructures.

For a thin film with a thickness of approximately 20 nm:

A 1 wt/vol% p(DMS-co-AMS) copolymer was dissolved in *o*-xylene with ≈5 wt/wt % (respective to the copolymer) of the photoacid generator (triphenyl sulfonium triflate). The solution was left to dissolve overnight. To enable coating onto a Si wafer, the solution was filtered through a 0.45 micron PTFE filter and spin coated at ≈2000 rpm for ≈60 s. Following this, the wafer was heated on a hotplate at 120 °C for 60 s. The wafer was irradiated with a broad band UV flood lamp for ≈20 min (wavelength 365 nm) using a foil mask. Following irradiation, the wafer was heated on the hotplate for 60 s at 120 °C. The thickness was then measured using an alpha-SE^®^ ellipsometer (Lincoln, NE, USA). To test crosslinking, the film was washed with toluene, heated on a hotplate to remove the solvent, and the thickness measured once more by ellipsometry. The thickness was kept unchanged to ensure that the crosslinking reaction worked well. Subsequent pyrolysis of the copolymer on the wafer was carried out in a furnace at 1300 °C under nitrogen (10 °C/min heating rate), as shown in Figure 1.

In the second part of the current work, the nano-sized filler particle of Al_2_O_3_ was dissolved in isopropanol, although the solvent for the other precursors was xylene. The concentration of all the precursor solutions was 50 mg/mL. After combination under ultrasonic agitation for 30 min to obtain homogenous and stable dispersion, these solutions were then filtered through a PTFE filter, and the procedure for the coating was roughly the same as for the unfilled Al_2_O_3_ copolymer. Pyrolysis was carried out in a horizontal alumina tube furnace under a helium purge, and the heating rate employed was 10 °C/min from room temperature to 900, 1000, 1100, 1200, and 1300 °C.

### 2.4. Characterizations

#### 2.4.1. Thickness Measurement

Thickness alterations in the process of thin-coating the copolymer film were estimated using a spectroscopic alpha-SE^®^ ellipsometer (J.A. Woollam Co., Lincoln, NE, USA). Si wafers were employed as the substrates in this estimation. The set of frameworks and test procedures were as follows: the wavelength ranged from 380 to 890 nm, and both 65° and 70° were used as angles of incidence. The thicknesses of layer films were determined using the Cauchy model, with A*_n_* and B*_n_* as the fit parameters set at 1.45 and 0.01, respectively. Then, the thicknesses of the layer films were automatically calculated.

#### 2.4.2. Fourier Transform Infrared (FT-IR) Spectroscopy

The characteristic absorption spectrum of a thin PDMS-copolymer film was characterized by FT-IR spectrum one. The spectra were recorded from a wavelength of 525 to 4000 cm^−1^, a number of scans equal to 32, and a resolution of 4 cm^−1^. The spectra were manipulated with Spectrum software (PerkinElmer FT-IR spectrometers, Norwalk, CT, USA).

#### 2.4.3. X-Ray Diffraction (XRD)

X-ray Diffractometer (JEOL JDX-8030, Tokyo, Japan) was used to analyze the phase structure of the coatings. Cu Kα radiation of wavelength λ = 0.15418 nm was generated at 40 kV and 30 mA. Samples were scanned at 2°/min in a range of 2θ = 10°–80° using a step size of 0.1°.

#### 2.4.4. Thermal Analysis (TGA/DSC)

The thermal analyses were performed with a simultaneous TGA-DSC (SDT Q600, TA Instruments, New Castle, DE, USA) under an inert atmosphere of helium at a heating rate of 10 °C from room temperature to 900 °C to 1300 °C.

#### 2.4.5. Scanning Electron Microscopy (SEM)

The surface morphology of the composite coatings was observed by SEM (JEOL JSM-IT300, Tokyo, Japan). In addition, the SEM was equipped with an energy-dispersive X-ray spectroscopy module (EDS, Oxford, UK) for surface element analysis.

## 3. Results and Discussion

### 3.1. Spectroscopic Ellipsometry Measurement

To quantify the covering thickness developing on the substrates, spectroscopic ellipsometry data were obtained with precision to the nanoscale. As seen in Table 1, the thickness of p(DMS-co-AMS) was 18.1 ± 0.1 nm, which demonstrates the achievement of pretreatment. At that point, the thickness expanded to 20.8 ± 1.4 nm after the preceramic polymer was loaded with nano-sized Al_2_O_3_. It is difficult to achieve such a high thickness in a self-assembly monolayer or a physically adsorbed film. Aside from hydrophilicity, topography change is another critical factor influencing the antiadhesion properties of the material surface [15]. The copolymer part was observed to be exceptionally effective at scattering the hydrophilic Al_2_O_3_ nano-sized powders into the exceedingly hydrophobic solvent [16,17].

### 3.2. Fourier-Transformed Infrared Spectroscopy

**Figure 2:**
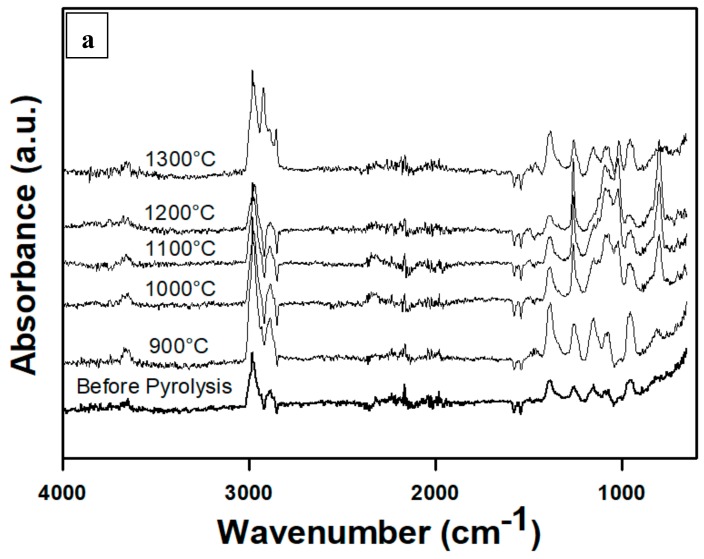

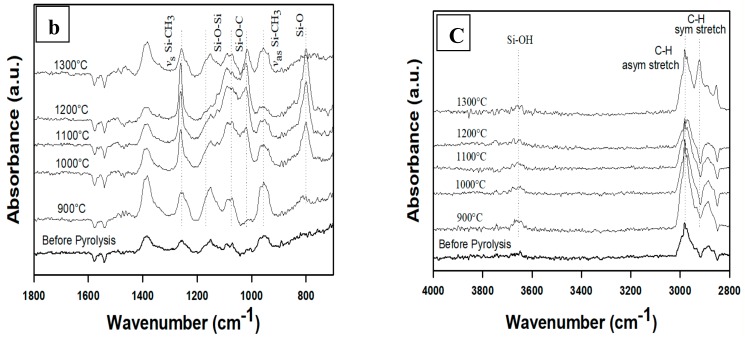
FTIR spectra of p(PDMS-co-AMS) copolymer, pyrolyzed between 900 and 1300 °C (**a**) Full spectrum from 4000-600 cm^−1^; (**b**) Partial spectrum from 1800-700 cm^−1^; (c) Partial spectrum from 4000–2800 cm^−1^.

Figure 2 introduces the FT-IR spectra for the p(PDMS-co-AMS) copolymer. The characteristic bands of Si-O and Si-C are conspicuous in all the spectra. The SiC shapes are formed at relatively higher pyrolyzing temperatures; however, the process begins with the formation of the SiO_4_ mixed bonds (which are mostly the building blocks of the Si-OC phase). The observed bands include a Si–O–Si rocking vibration, which occurs at 455 cm^−1^; and an O–Si–O band bending which appears at 790 cm^−1^ and overlaps with Si–C stretching vibrational band which occurs at 830 cm^−1^. The Si-C vibration leads to the observed weak absorption at 620 cm^−1^. Additionally, within the 1000–1060 cm^−1^ range comes Si–O. The fact that Si–O bonds are sparsely distributed in the SiO_4_ coordination affirms the move to move to convey down wavenumbers [18,19,20,21].

The power of the Si–O groups occurring at 800 cm^−1^ and 1000–1600 cm^−1^ is sharply reduced, which can be attributed to the increasing temperature of pyrolysis. It is worthwhile noticing that the band at 800 cm^−1^ completely disappears at the beginning of the pyrolysis process. A huge intensity increment, and the move of the overlying SiO and the Si-C vibration which is at 960 cm^−1^, occur at the same time. As the temperature rises to 900 °C, the Si-O vibration persists irresistibly such that its characteristic absorption becomes dominant at 950 cm^−1^. Comparing this observation with a sample pyrolyzed at 1300 °C, the Si–O peak observed above shifts to 1120 cm^−1^, further indicating a considerable reduction in the Si-O contribution, and instead, a substantial increment in the vibration of Si-C. Considering temperatures 1000, 1100, and 1200 °C, there is only the Si-C vibration occurring at 1080 cm^−1^ that can be distinguished. The progressive degradation of the Si-OC network (as shown by the vanishing Si-O intensity and powerful Si-C vibration) is mapped by the gradual improvement of the Si-O and Si-C absorption bands with pyrolysis temperature. The findings are in harmony with the findings from XRD in terms of the Si-C crystallization and Si-C domain growth for samples at 1200 and 1300 °C: a strong C-H symmetric and C-H asymmetric stretching vibrations (2920–2985 cm^−1^) and a weak peak at 3650 cm^−1^ (Si-OH). These peaks agree fairly well with those of Li et al. [16] and Plummer et al. [22]. Finally, in order to identify the bands situated at 1395 and 1460 cm^−1^ for the 1300 °C sample, symmetric deformation vibration of Si-CH_3_ group and the asymmetric deformation band for Si–CH_3_ can aid in such identification [23]. 

**Figure 3:**
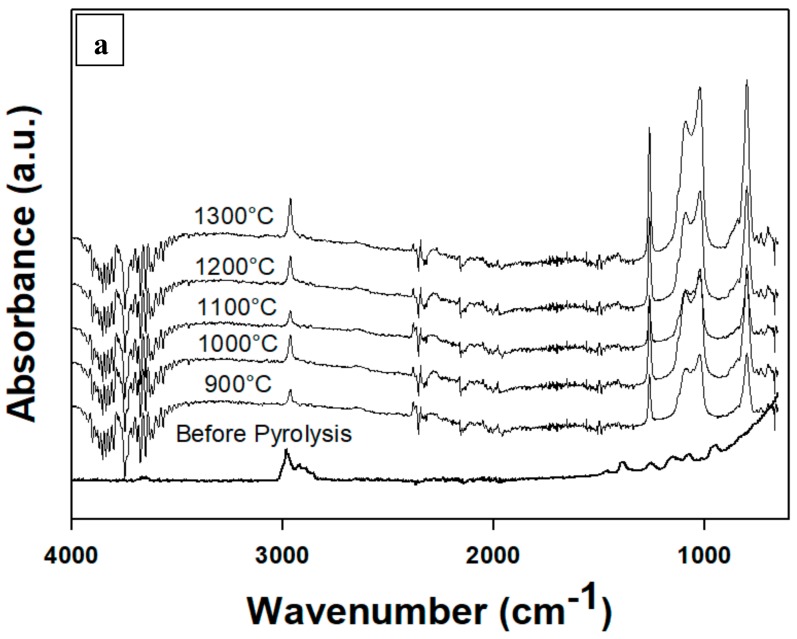

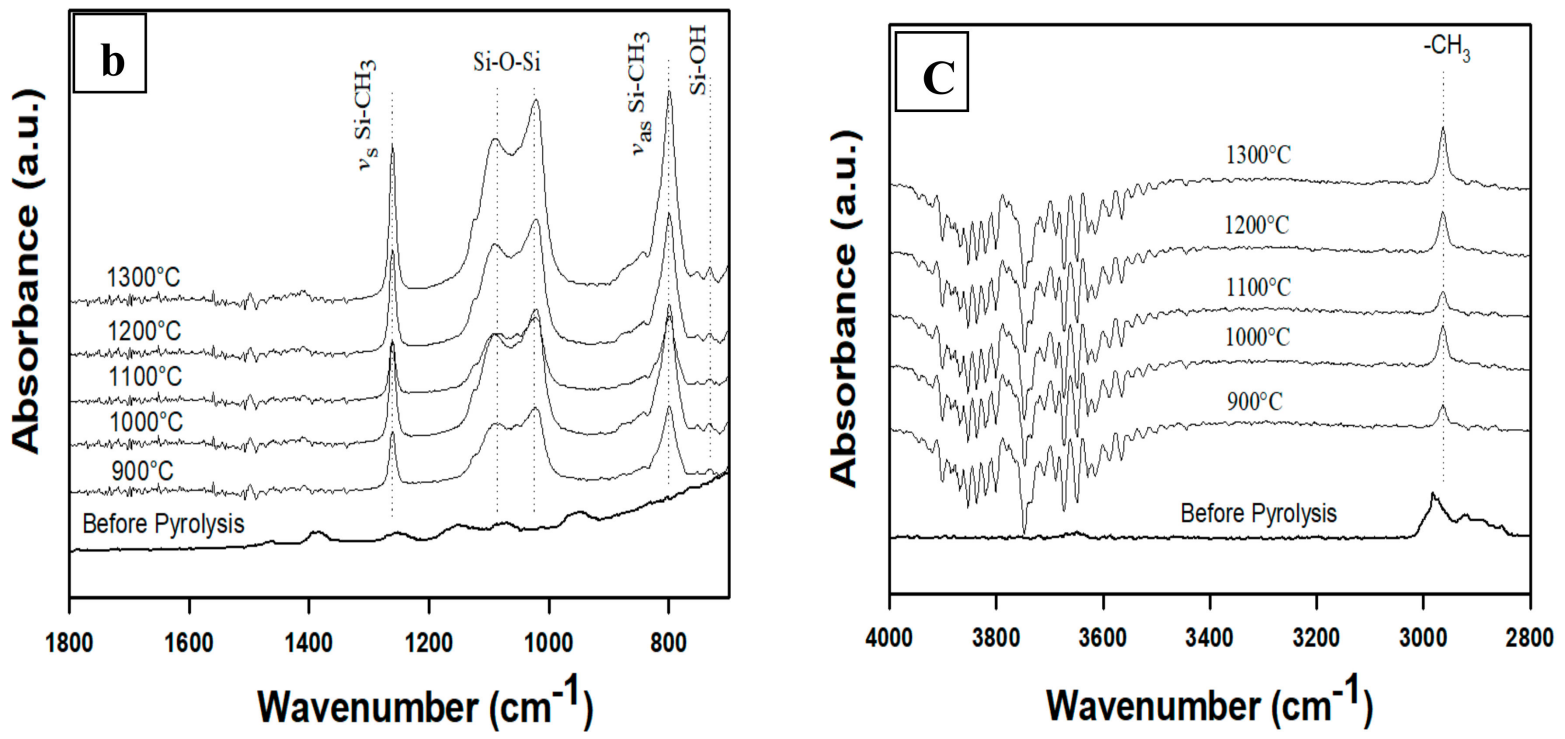
FTIR spectra of Al_2_O_3_/p(PDMS-co-AMS) copolymer, pyrolyzed between 900 and 1300 °C. (**a**) Full spectrum from 4000–600 cm^−1^; (**b**) Partial spectrum from 1800–700 cm^−1^; (c) Partial spectrum from 4000–2800 cm^−1^.

When ceramic materials, i.e., Al_2_O_3_/p(PDMS-co-AMS), were subjected to FT-IR measurements, Si–O–Si units were found (absorption band at 959 cm^−1^; see Figure 3). The spectrum of pyrolyzed materials demonstrates absorption bands at 590 cm^−1^ (SiOC–H vibration), 788 and 1258 cm^−1^ (Si–CH_3_ vibration), and 1122 cm^−1^ (Si–O–Si vibration), as previously reported [24,25], were present. The vibrational modes attached to Si-O and Si-C were clear from the FT-IR spectra plotted (Figure 3) that came from the SiO_4−*x*_C*_x_* (*x* = 1–4) combined bonds of Si-O-C and Si-C, together with methyl C-H vibration at 2970 cm^−1^ [16]. An increase in temperature leads to decreased vibrations of the Si-O when compared with Si-C bonds. This explains why the carbothermic reaction of silicon degraded the Si-O network and corresponded to the results of the XRD analysis [23]. However, the bands ascribed to Si-OH group at about 3700 cm^−1^ are undetected in the spectrum of Al_2_O_3_/p(PDMS-co-AMS).

### 3.3. X-Ray Diffraction

**Figure 4:**
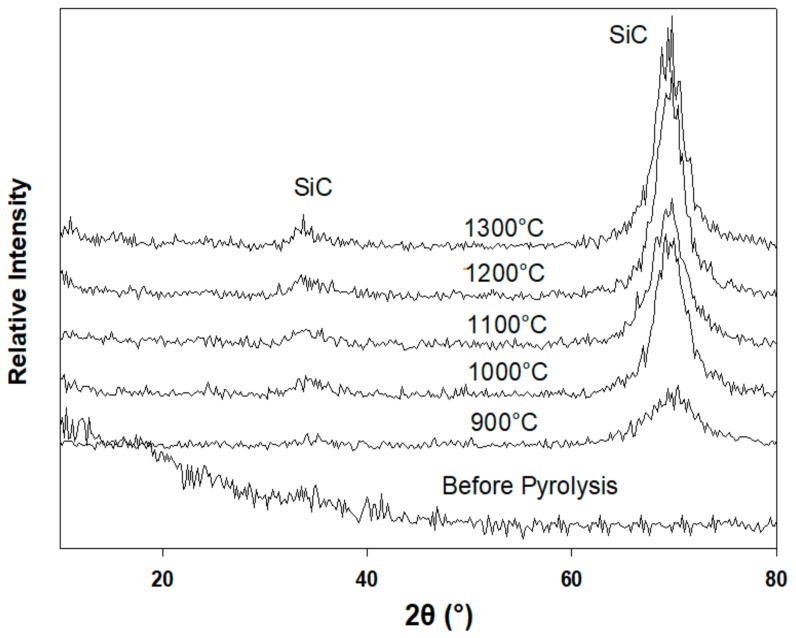
X-ray diffraction patterns of p(PDMS-co-AMS) copolymer, pyrolyzed between 900 and 1300 °C.

The p(PDMS-co-AMS) copolymer samples pyrolyzed at 900, 1000, 1100, 1200, and 1300 °C were explored for their crystalline phase compositions by means of a thin-film XRD (Figure 4). Each sample prepared prior to pyrolysis was observed to be X-ray amorphous. The p(PDMS-co-AMS) produced at 1300 °C revealed the presence of β-Si–C nanocrystallites with diffraction reflexes at 2θ = 34.7° (111) and 70.0° (311) and having a size of 1–2 nm (as determined by the Debye–Scherer equation). Moreover, a turbostratic graphitic-like phase was observed to be available in the sample, with a fundamental diffraction reflex at 2θ ≈ 43° [26]. 

**Figure 5:**
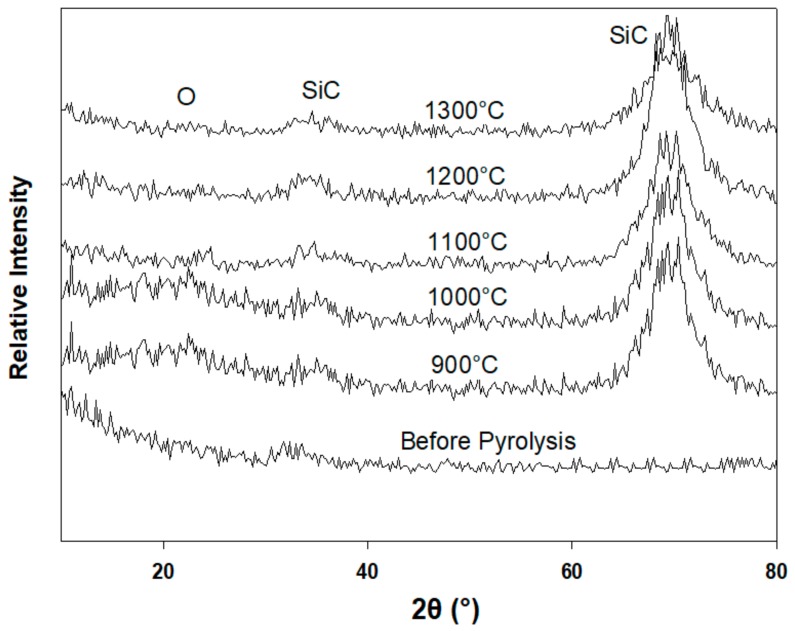
X-ray diffraction pattern of Al_2_O_3_/p(PDMS-co-AMS) copolymer, pyrolyzed between 900 and 1300 °C.

The XRD studies indicate a gradual increase in the amount of crystalline Si–C. Figure 5 demonstrates the XRD patterns of the pyrolyzed thin films compared with the unpyrolyzed sample of the Al2O3/p(PDMS-co-AMS) copolymer. The broad peak close to 22° is credited to the short-range order in amorphous silica. The broad peak around 26° for the samples prepared at 1300 °C and higher can be identified thanks to the overabundance of free carbon, which shows up as turbostratic carbon. The third peak at 35.5° can be identified with β-SiC. The broadness of the peak suggests the nanoscale dimensions of the β-SiC crystallites. Despite the fact that the amount of the Si–C phase increased at higher temperatures, the related peak remained broad. This is because of limited crystal growth as a result of the poor diffusivity of species in Si–OC. Estimation of the crystallite size of Si–C as per the Scherer equation reveals a minimal development from 1 nm at 1100 °C to around 3 nm at 1300 °C.

### 3.4. Scanning Electron Microscopy

**Figure 6:**
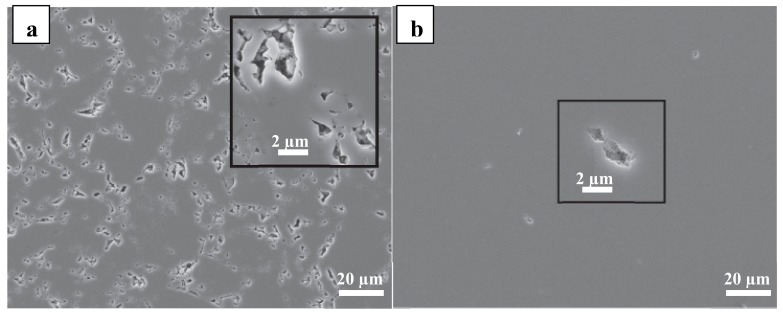
SEM images showing the pores in the samples pyrolyzed at 1100 °C (**a**) and 1200 °C (**b**).

Figure 6a,b illustrates SEM pictures of p(PDMS-co-AMS) copolymer after being pyrolyzed at 1100 and 1200 °C, respectively. The necks of the polymer were well-shaped when pyrolyzed at 1100 °C, but the microstructure remained porous. The pores vary in size, but the largest is usually about 20 microns in its diameter. After being heated at 1200 °C, the polymer became denser and was visible. At this temperature, porosity decreased considerably, and the most significant pore reduced to about 5 mm. However, the morphology and volume fraction did not change. Further analysis was needed to examine the system of densification between pyrolysis at both temperatures. At high temperatures, some hydrogen was retained, and this contributed to densification. Electrical resistance at the boundaries of the particles resulted in overheating and softening. This enhanced the creep formation as temperatures increased, thus increased densification. These are the explanations for the rapid elimination of pores at the high temperatures of 1100 and 1200 °C.

**Figure 7:**
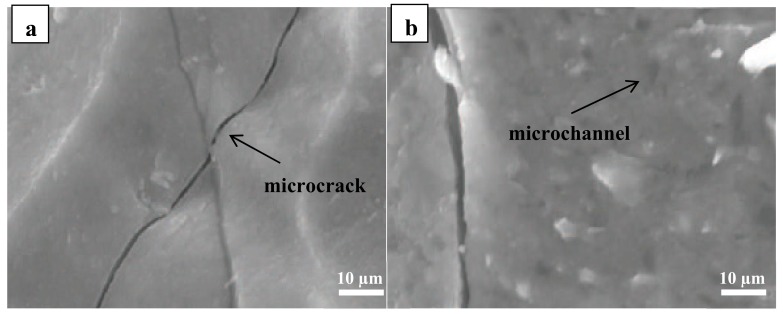
SEM images of samples pyrolyzed at 1300 °C. Note that the regions between the microcracks (**a**) and microchannels (**b**) do not reveal any indication of closed porosity. Hence, the regions between such structural defects are considered fully dense.

More details on the microstructure were acquired from the SEM examination. The SEM micrographs of the p(PDMS-co-AMS) copolymer at 1300 °C are shown in Figure 7. The connection between microcracks, gas channels, and nearby crystallization shows that the latter is related to the grain coarsening observed. Although the appearance of the microcracks can be attributed to the advancement of gases during the ceramization procedure, which can be suppressed by further expanding the cross-connecting level of the precursors and additionally changing the pyrolysis parameters, the appearance of extensive cracks can be identified by the thickness of the prepared samples. The relationship between thickness and crack formation still needs further investigation; nevertheless, the thermal stability of these materials, as presented below, emphasizes their potential for high temperature applications, for example, as protective thermal coatings in oxidative environments.

**Figure 8:**
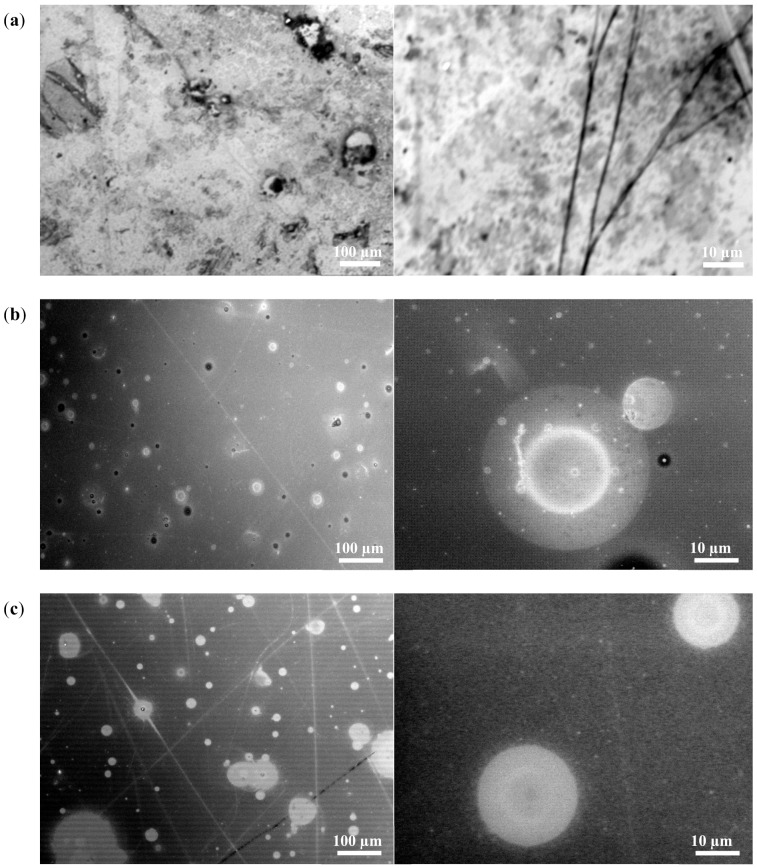

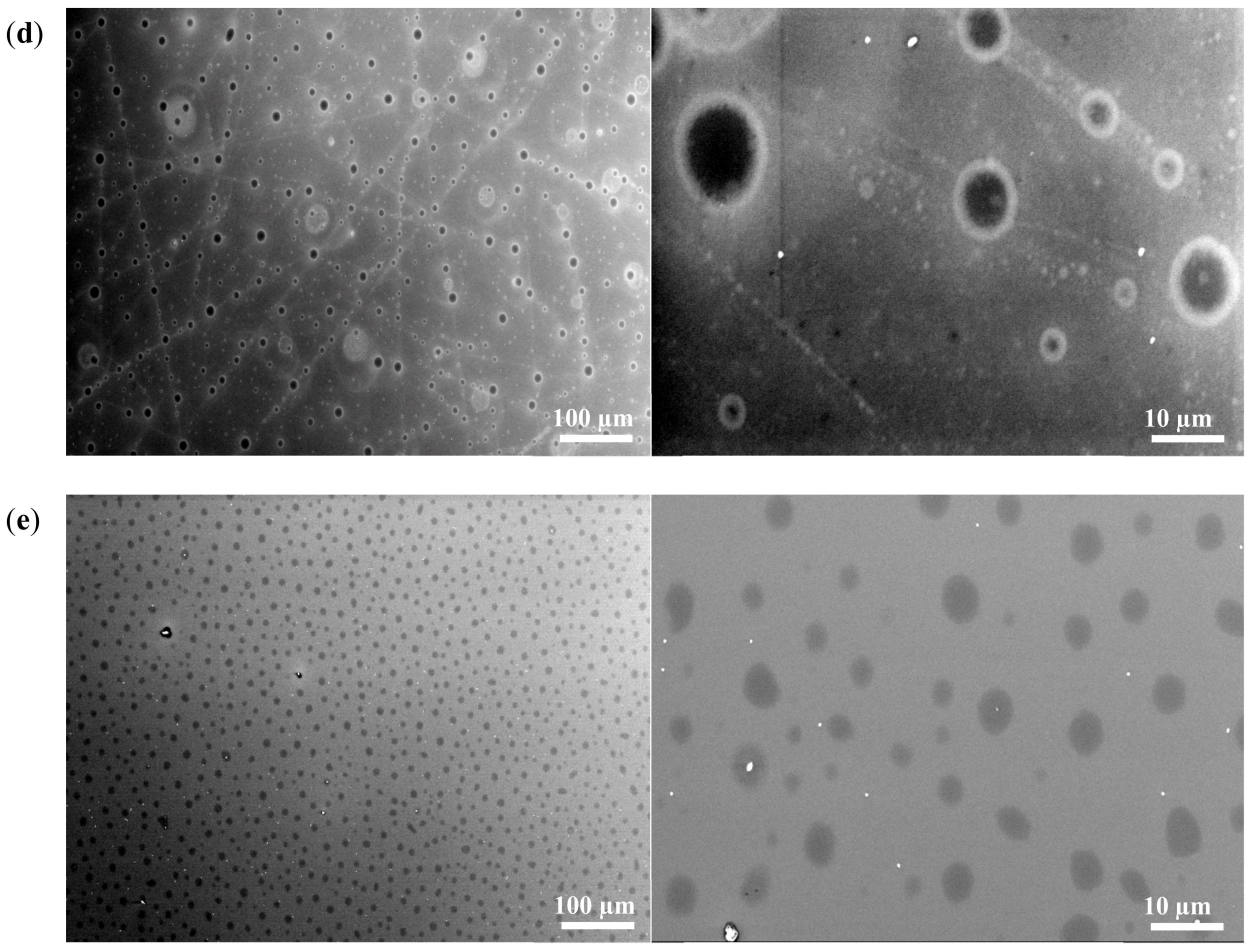
SEM images of samples Al_2_O_3_ filled copolymer: (**a**) 900 °C, (**b**) 1000 °C, (**c**) 1100 °C, (**d**) 1200 °C, and (**e**) 1300 °C. (Elemental distributions detected by SEM-EDX for the points labeled on the fracture surface image shown in the Supplementary Materials).

In terms of SEM imaging, the closed porosity system is merely negligible. Al_2_O_3_/p(PDMS-co-AMS) copolymer samples are known to contain micro-cracks, and at times, microchannels at a temperature as high as 900 °C. Bulk materials are, however, very dense despite the presence of large porosity between the microparticles (Figure 8). Therefore, in the discussion of density versus processing parameters, this issue of the non-existence of porosity is not allowed.

### 3.5. Thermogravimetric Analysis 

**Figure 9:**
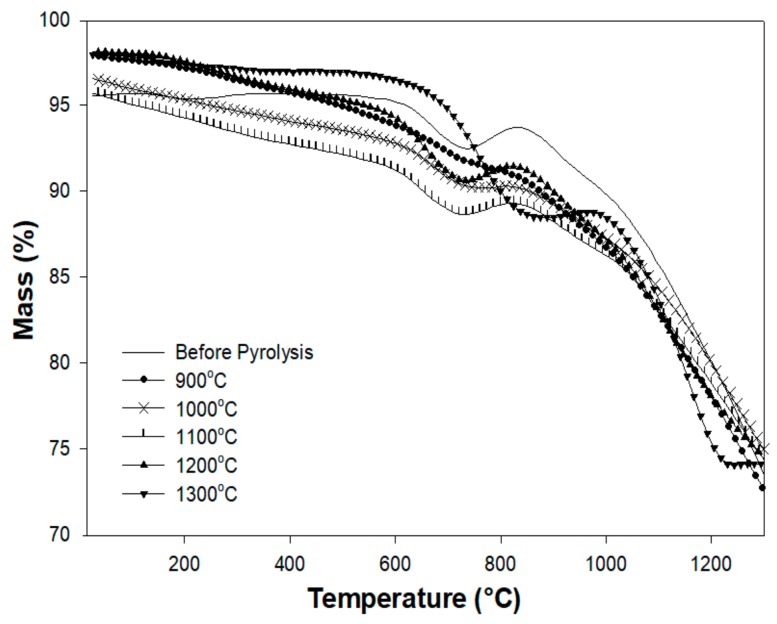
TGA curves of the pyrolyzed preceramic polymer between 900 and 1300 °C.

**Figure 10:**
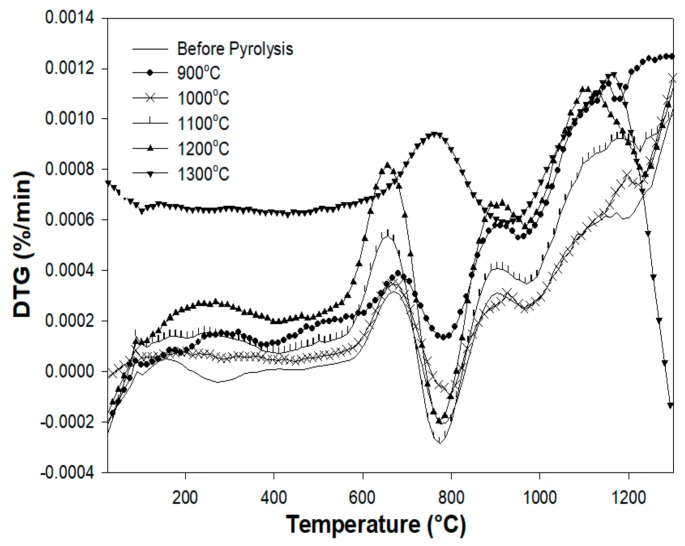
DTG curves of the pyrolyzed preceramic polymer between 900 and 1300 °C.

Figure 9 shows the TG curve of the Al_2_O_3_-filled copolymer prior to pyrolysis, and the pyrolyzed samples from ambient temperature to 1300 °C. The polymer-to-ceramic transformation induces a total weight loss of 25 wt %, which occurs in three main decomposition steps in the temperature region between 170 and 1100 °C, as shown by the derivative thermal gravimetry (DTG) curve (Figure 10). According to FT-IR studies, in the first step (temperature range from 170 to 370 °C, DTG peak at 255 °C), crosslinking procedures occur with the release of isopropanol. The IR spectrum of Al_2_O_3_/p(DMS-co-AMS) shows absorption bands at 790, 970, 1275, and 2950 cm^−1^, which were assigned to *v*_as_(Si–CH_3_), *v*_s_(Si–O–Si), *v*_s_(Si–CH_3_), and *v*_s_, *v*_as_(C–H), respectively [27].

In the second step (from 650 to 900 °C, DTG peak at 785 °C), in the 600–800 °C range, methane is the main volatile species being released. Furthermore, the evolution of hydrogen and methane was found in the second decomposition step by means of in situ IR spectroscopy and MS, indicating the start of the ceramization process [28]. In the last step (temperatures from 900 to 1100 °C, DTG peak at 1000 °C), ceramization is completed, and the release of hydrogen is observed.

The pyrolysis mechanism involved in the polymer-to-ceramic conversion is generally quite complex. This reaction sequence involves structural rearrangements and radical reactions that result in the cleavage of chemical bonds (e.g., Si–H, Si–C, and C–H), the release of organic functional groups (e.g., CH_4_), and the formation of an inorganic network [1]. This process could be schematized as a sequence of different distinct rearrangement reactions, with each one being characterized by its specific temperature range.

Thermal decomposition during pyrolysis is another fundamental factor, because it represents the step in which most of the weight loss is observed, which is derived from the cleavage of chemical bonds and the release of gaseous by-products. Finally, dehydrogenation and carbothermal reduction reactions at higher temperatures could give an additional contribution to the global weight loss, although these phenomena are generally less significant [28].

If the surface-to-volume ratio of the green body is too low, the removal of the gaseous by-products can be hindered, resulting in an interior pressure increase and crack formation. Furthermore, it should be noted that despite the relatively high mass loss of the polymer during ceramization, the residual porosity of the ceramic parts is negligible.

### 3.6. Differential Scanning Calorimetry

**Figure 11:**
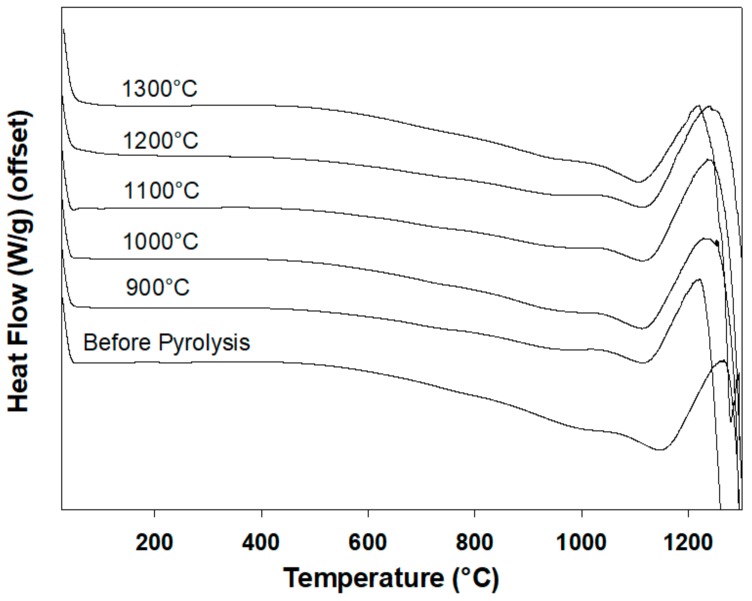
DSC heating thermograms obtained with the samples indicated on the plot at a heating rate of 10 °C/min.

DSC heating thermograms of Al_2_O_3_/p(PDMS-co-AMS) copolymers prior to and after pyrolysis are shown in Figure 11. Each curve is offset to allow for a comparison between the different samples. We observe the glass transition (T_g_) at around 900 °C and a single endothermic melting peak (*T*_m_) at around 1100 °C (Table 2). The T_gs_ for SiOC ceramics show no significant difference in the copolymer prior to pyrolysis. The broadened transitions for the ceramics suggest cooling may be occurring, and hence resulting in the nucleation of crystallites [29]. The reductions of both the ceramics’ melting points (*T*_m_) (taken as the lowest point along the transition) and the total heat of melting (Δ*H*_m_) clearly show that the crystallization of the ceramic materials obtained on pyrolysis is not completely suppressed on the experimental time scales.

Stabilization of the amorphous state of PDCs is enabled by increasing the number of components in the Si-C systems [29]. Monthioux and Delverdier [29] did some research about the crystallization patters of several PDCs. In their study, they concluded that the nucleation of excess free carbon commonly found in PDCs is usually the initial phenomenon to occur, followed by the nucleation of Si-C in the crystallization procedure. The temperatures at which different materials crystallized were observed. Based on this, some compounds were found to crystallize at temperatures as low as 900–950 °C, but the ternary crystallization of Si-CO systems was observed at 1100–1250 °C. The quaternary Si-C-N-O system did not crystallize even at temperatures as high as 1400 °C. An increase in the number of components in polymer-derived ceramics was not the only contributing factor to an increase in stability against crystallization. Other factors included chemical composition, glass architecture, residual porosity, and starting polymer.

## 4. Conclusions

Preceramic polymers have been recognized as a critical tool in the production of advanced ceramics [1,2,3,4,5,6,7]. This work focused on polysiloxanes and aluminum oxide nanoparticles. The findings of the work indicate that a combination of the lithography and pyrolysis properties of polymers can produce quality ceramics. This is depicted in the analysis of the conversion of the p(DMS-co-AMS) copolymer to ceramic material using photolithographic application, herein leveraging the pyrolysis properties of the polymers, which change with changing temperature as they impact the chemical rearrangement reactions to form ceramics. The results show that the p(PDMS-co-AMS) copolymer has excellent pyrolysis properties for self-assembled UV lithography applications. The thermal decomposition patterns of the final ceramics at different temperature levels suggest particular thermolysis pathways, which are regulated kinetically or thermodynamically. Additional chemical reactivity was observed when the ceramic amorphous products were heated at 900 °C and crystallized at 1300 °C.

## Figures and Tables

**Table 1:** A summary of the thicknesses measurements of p(PDMS-co-AMS) and Al_2_O_3_/p(PDMS-co-AMS) copolymer coatings.

	p(PDMS-co-AMS)	Al_2_O_3_/ p(PDMS-co-AMS)
Thickness (nm)	18.1 ± 0.1	20.8 ± 1.4

**Table 2:** Parameters of the glass transition determined by DSC: glass transition temperature (T_g_), onset and end temperatures (T_on_, T_end_), and melting temperature (T_m_).

Sample	T_g_ (^°^C)	T_on_ (^°^C)	T_end_ (^°^C)	T_m_ (^°^C)
Before Pyrolysis	978	871	1020	1146
900^°^C	898	838	960	1116
1000^°^C	922	834	987	1111
1100^°^C	905	844	963	1122
1200^°^C	890	830	933	1119
1300^°^C	919	846	961	1114

## Supplementary Materials

Supplementary materials can be accessed at https://www.mdpi.com/1996-1944/13/5/1140/s1.
