# Supplementary material for: Synthesis Based on a Preceramic Polymer and Alumina Nanoparticles via UV Lithography for High Temperature Applications
Source: Materials (Basel). 2020 Mar 4;13(5):1140. doi: 10.3390/ma13051140 (PMC7084998; doi:10.3390/ma13051140)
Supplement: Supplementary file 1 [file materials-13-01140-s001.pdf]

Dear Dr. Amber Zhu,

We would like to thank you and the reviewer(s) for handling of our manuscript and insightful comments to improve the readership. Below is the response to the comments, the **red font** in the main text referring to the modified content.

Reviewer # 1:

Comments to the Author

The article that I received for review raises an important problem of new method to produce ceramics based on combination of lithography and pyrolysis properties of polymers. The title well reflects manuscript content and properly reflects topic of the presented investigation. The manuscript is well written in general. The experimental and discussion parts are understandable and the results make sense. An abstract appropriately indicates the experimental approach and used methods. The Introduction is acceptably long and detailed to provide a general outline to a concept of interest. The experimental chapters are well written. Conclusions in Summary are clear and adequate. References are sufficient. I appreciate the efforts of the Authors and admire the multitude of the used research methods. However, the Authors did not avoid an some understatement. The following problem arouse my doubts:

The Authors have written

“It is very characteristic for Al2O3/p(PDMS-co-AMS) copolymer samples to contain microcracks and sometimes microchannels; the former are observed in the 900°C samples. However, it should be noted that the bulk material between such open porosity (cracks and pore channels) is, without exception, fully dense (Figure 8). Therefore, the presence of closed porosity is excluded in theoverall discussion of density versus processing parameters.”

In my opinion, the existence or absence of closed porosity cannot be excluded solely on the basis of microstructural tests (SEM micrographs).

In my opinion the article includes innovative results and could be of interest to the readers. I recommend it for publishing after minor corrections.

Response to Reviewer #1

In the Results and Discussion

The following information is included in 3.4 Scanning electron microscopy (page 11, line 256-257).

In terms of the closed porosity of this system, it is proposed based on SEM imaging that it is generally negligible. It is very characteristic for Al2O3/p(PDMS-co-AMS) copolymer samples to contain microcracks and sometimes microchannels; the former

are observed in the 900°C samples. However, it should be noted that the bulk material between such open porosity (cracks and pore channels) is, without exception, fully dense (Figure 8). Therefore, the presence of closed porosity is excluded in the overall discussion of density versus processing parameters.

Reviewer #2:

Comments to the Author

Almeataq et al. clearly describe the ceramization process of preceramic polymer via UV curing and heat treatment. I just noticed some minor issues in the manuscript. After the authors address them, the paper can be published with no doubt.

1. How to confirm the grain size as claimed by the authors to be 1-3 nm?
2. Where should the Al peak be in Figure 5?
3. On page 9, why do the author mention electric field? Is E field applied during the process?
4. SEM images in Figure 8 are too dark to see.

Response to Reviewer #2

Response 1: The crystallites with a size of 1-3 nm are calculated by Debye-Scherer equation. Future investigations will aim to confirm this issue by means of transmission electron microscopy and Raman spectroscopy.

Response 2: XRD results did not show peaks of Al in Figure 5. The EDX view of Al gave approximately 0.1 wt% (Please see the appendix below for elemental composition by EDX).

	C (wt.%)	Si (wt.%)	Al (wt.%)	O (wt.%)	In (wt.%)
Before Pyrolysis	20.54	77.35	0.09	2.02	
900°C	10.70	87.37	0.11	1.82	
1000°C	20.43	76.49	0.09	2.99	
1100°C	20.90	75.65	0.08	3.37	
1200°C	21.64	76.24	0.09	1.91	0.13
1300°C	22.65	72.71	0.09	4.55	

Response 3: The p(DMS-co-AMS) layer is coated onto p-type silicon substrate using a spin coater at 2000 rpm. In future studies, a perpendicular electric field will be produced by applying a potential difference between the p(DMS-co-AMS) layer coated on Si wafer and a parallel plate ITO conductor with water trapped between them.

Response 4: The SEM images in Figure 8 are adjusted by Adobe® Photoshop CS6 to correct the photo lighting (page 13-14).

Appendix

Elemental distributions detected by SEM-EDS for the points labeled on the fracture surface image shown in Figure 8.

Examples of these data are included in the Supporting Information as **Table 1**.

	C (wt.%)	Si (wt.%)	Al (wt.%)	O (wt.%)	In (wt.%)
Before Pyrolysis	20.54	77.35	0.09	2.02	
900°C	10.70	87.37	0.11	1.82	
1000°C	20.43	76.49	0.09	2.99	
1100°C	20.90	75.65	0.08	3.37	
1200°C	21.64	76.24	0.09	1.91	0.13
1300°C	22.65	72.71	0.09	4.55	

Before Pyrolysis

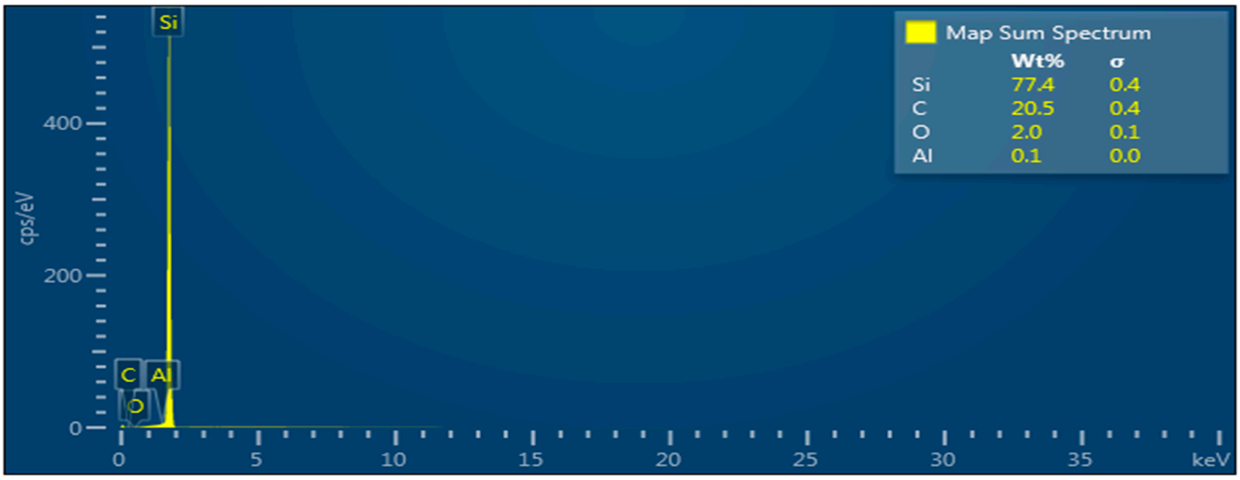

EDS Layered Image 1

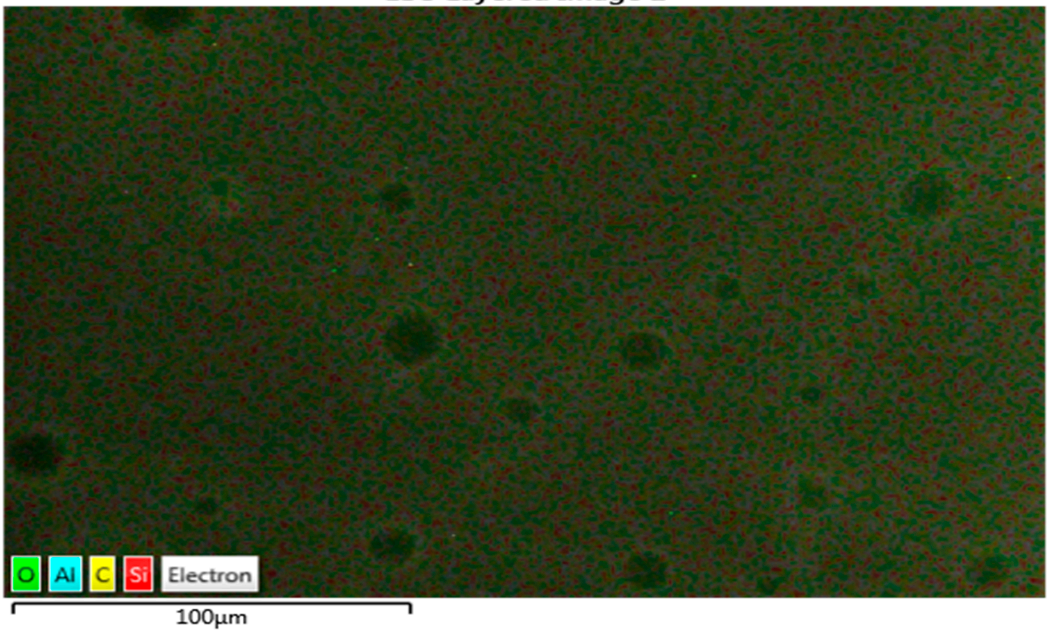

Element	Wt%	Wt% Sigma
C	20.54	0.37
O	2.02	0.06
Al	0.09	0.01
Si	77.35	0.37
Total:	100.00	

900°C

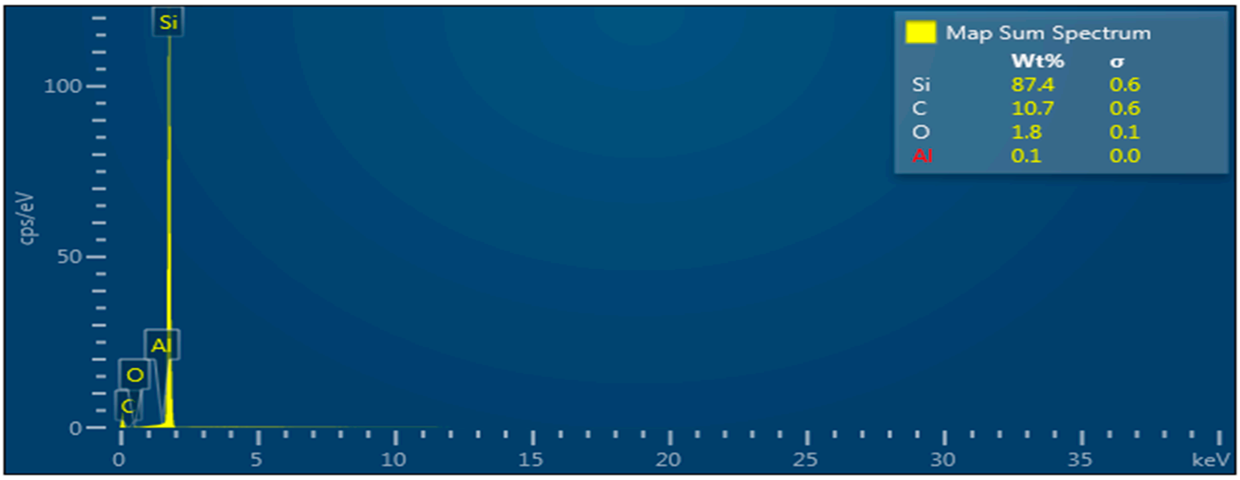

EDS Layered Image 1

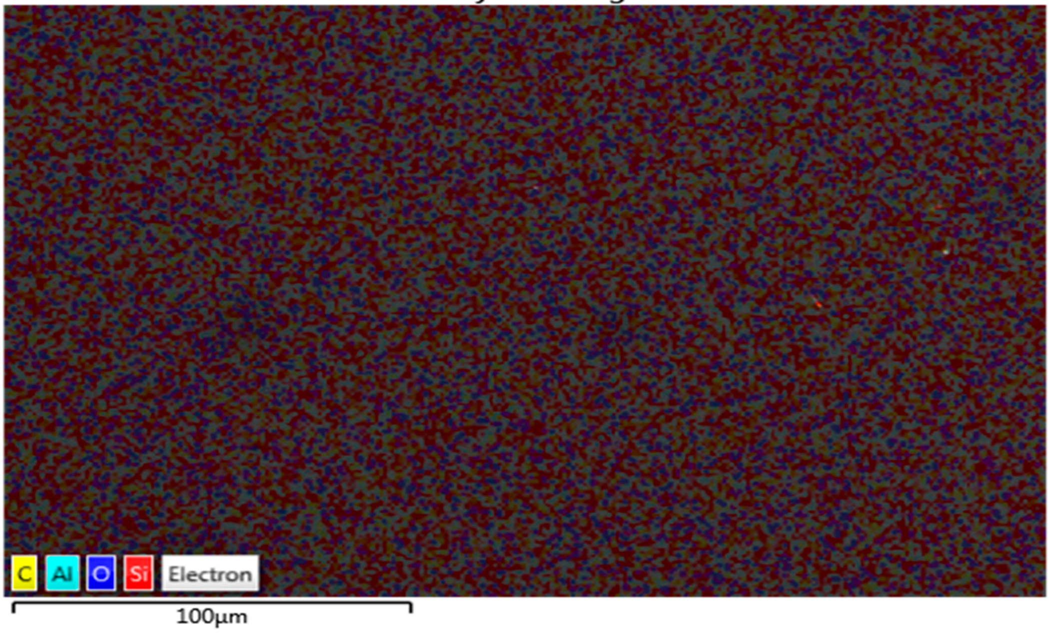

Element	Wt%	Wt% Sigma
C	10.70	0.56
O	1.82	0.10
Al	0.11	0.01
Si	87.37	0.55
Total:	100.00	

1000°C

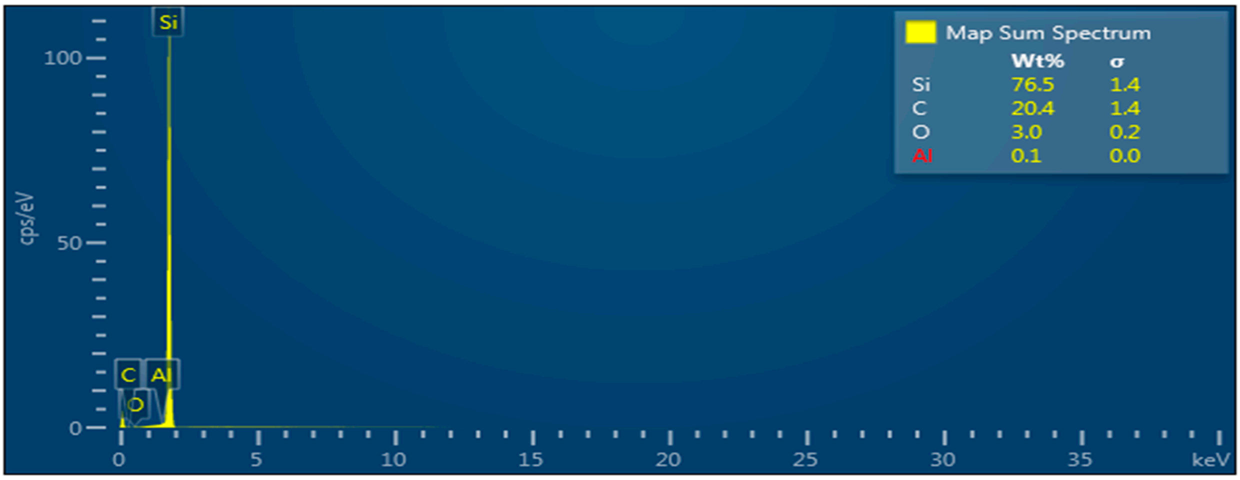

EDS Layered Image 1

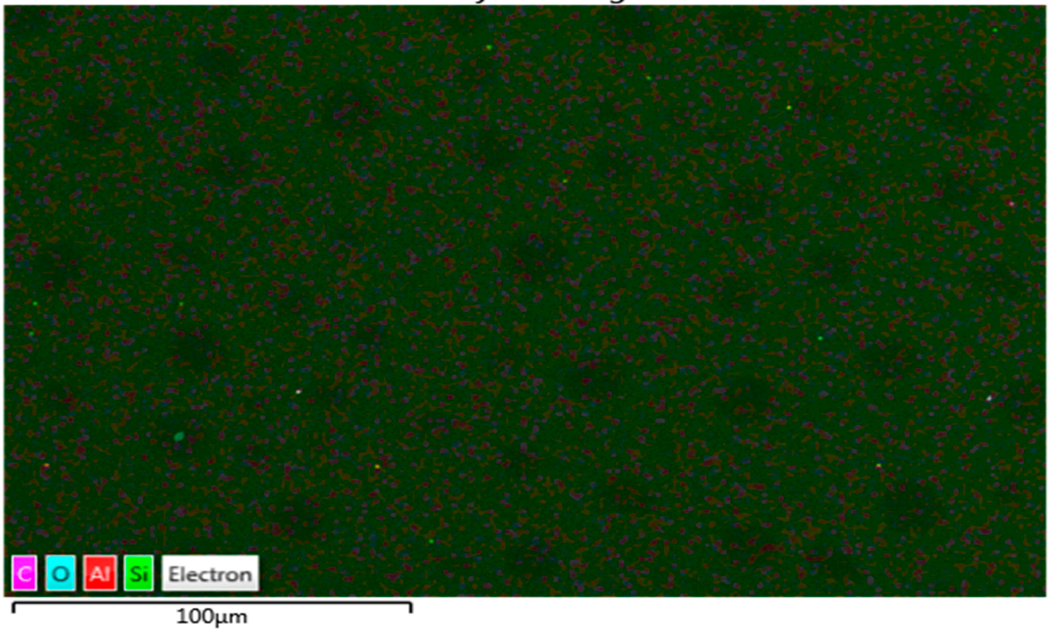

Element	Wt%	Wt% Sigma
C	20.43	1.40
O	2.99	0.22
Al	0.09	0.02
Si	76.49	1.35
Total:	100.00	

1100°C

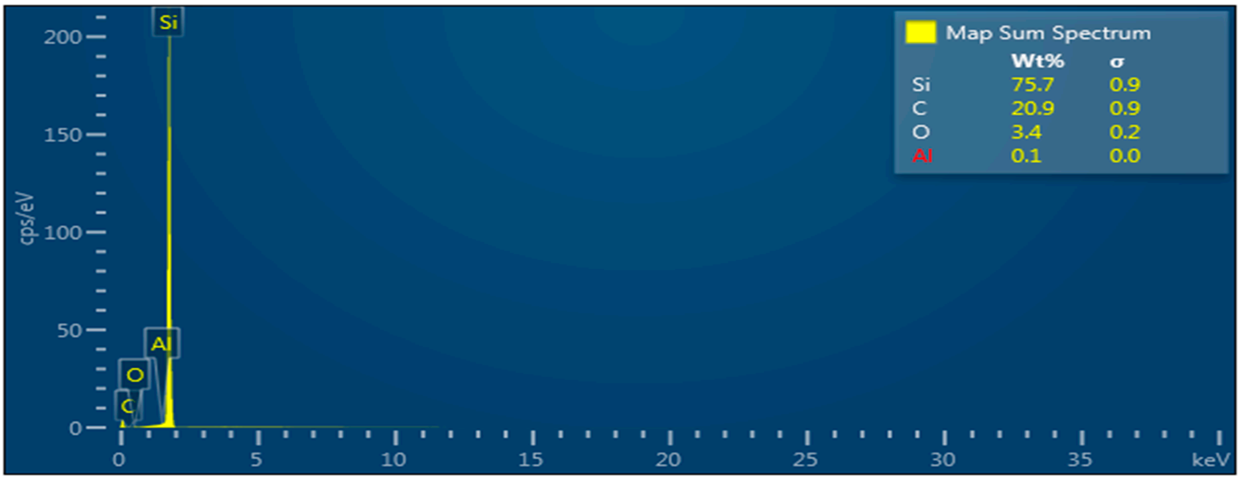

EDS Layered Image 1

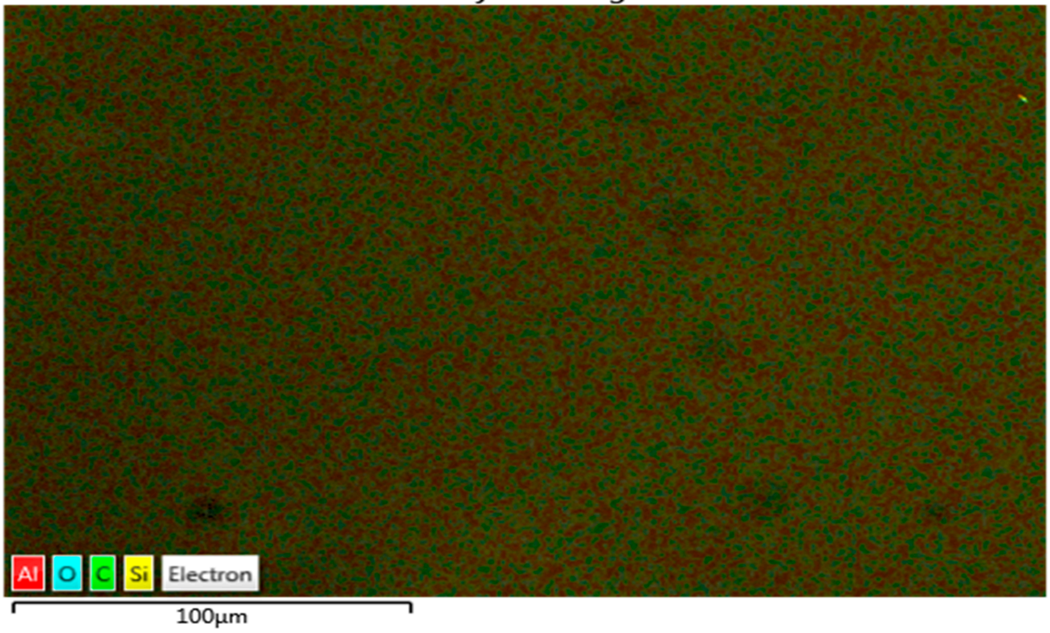

Element	Wt%	Wt% Sigma
C	20.90	0.90
O	3.37	0.15
Al	0.08	0.01
Si	75.65	0.86
Total:	100.00	

1200°C

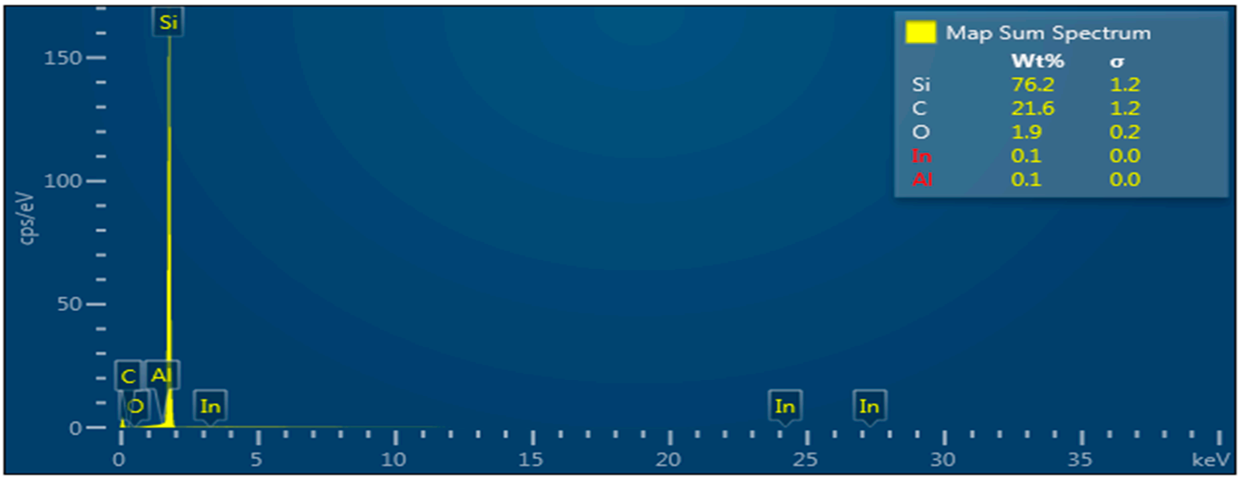

EDS Layered Image 1

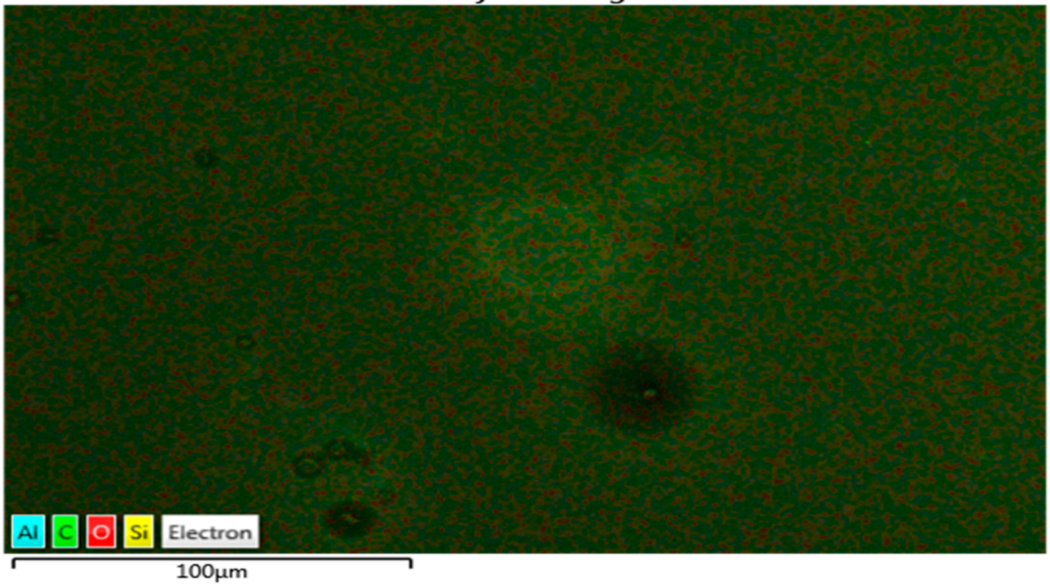

Element	Wt%	Wt% Sigma
C	21.64	1.21
O	1.91	0.15
Al	0.09	0.01
Si	76.24	1.18
In	0.13	0.02
Total:	100.00	

1300°C

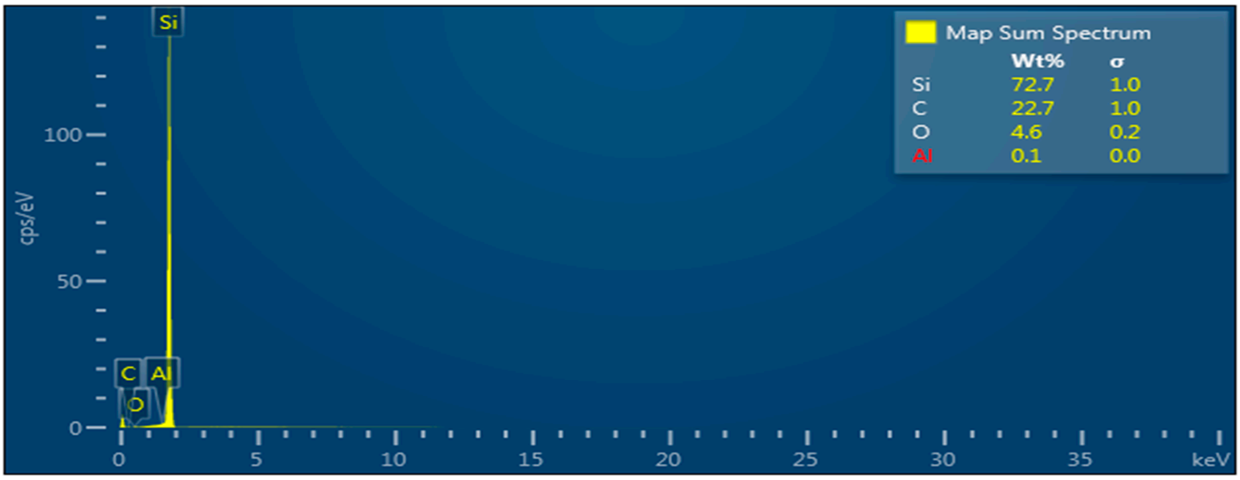

EDS Layered Image 1

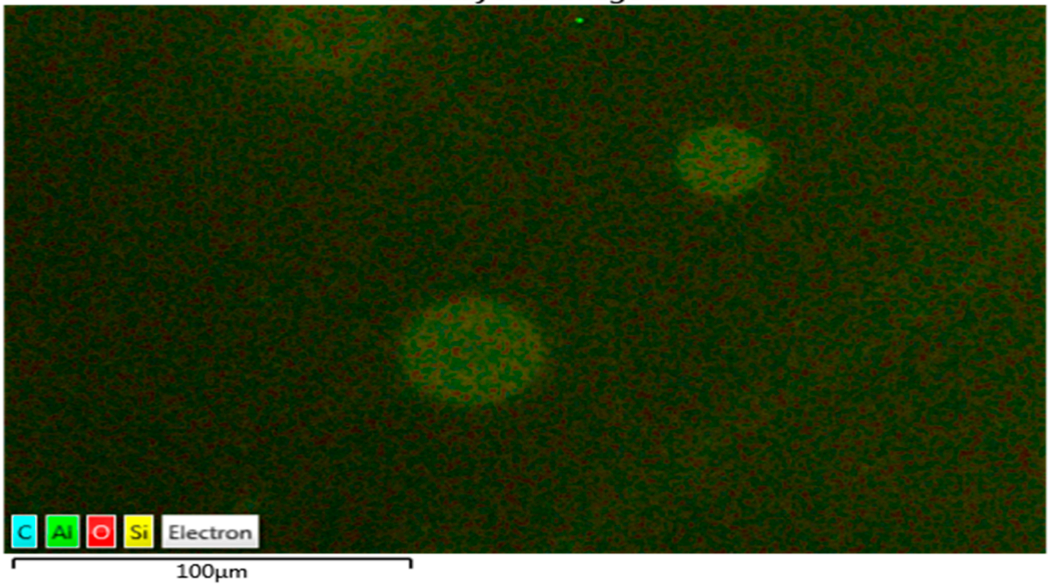

Element	Wt%	Wt% Sigma
C	22.65	1.03
O	4.55	0.18
Al	0.09	0.01
Si	72.71	0.98
Total:	100.00	
